# Association Between Household Income and Self-Perceived Health Status and Poor Mental and Physical Health Among Cancer Survivors

**DOI:** 10.3389/fpubh.2021.752868

**Published:** 2021-12-07

**Authors:** L. Joseph Su, Sarah N. O'Connor, Tung-Chin Chiang

**Affiliations:** ^1^Department of Epidemiology, Fay W. Boozman College of Public Health, University of Arkansas for Medical Sciences, Little Rock, AR, United States; ^2^Department of Environmental and Occupational Health, Fay W. Boozman College of Public Health, University of Arkansas for Medical Sciences, Little Rock, AR, United States

**Keywords:** health-related quality of life (HRQL), cancer survivorship, household income, mental health, physical health

## Abstract

**Background:** Health-related quality of life (HRQoL) is multidimensional and is composed of, at a minimum, self-perceived health status, physical functioning, and psychological well-being. HRQoL measures reflect the extent of disability and dysfunction associated with a chronic disease such as cancer. The objective of this study is to examine factors associated with HRQoL among cancer survivors.

**Methods:** Data from the 2009 Behavioral Risk Factor Surveillance System survey was used to examine factors associated with HRQoL among participants who reported having ever been diagnosed with cancer. Four questions associated with HRQoL included self-perceived health status, number of bad physical health days, and number of bad mental health days per month. Least square regression and logistic regression models, adjusted for confounding variables, were used for an ordinal and dichotomous [5 (bad) vs. 1–4 (excellent, very good, good, fair)] scale of HRQoL, respectively.

**Results:** Fifty nine thousand one hundred seventy three participants reported having ever been diagnosed with cancer. Adjusted mean self-perceived health status (5-point scale) among survivors of thyroid, colon, lung, cervical, breast, prostate, and ovarian cancer was 3.83 (0.05), 4.02 (0.04), 4.36 (0.06), 3.77 (0.03), 3.88 (0.03), 3.78 (0.04), and 3.96 (0.05), respectively. After adjusting for confounders, a positive dose-response effect was observed between income range and all three HRQoL measures across all seven cancer sites. Income was consistently and inversely associated with a higher chance for reporting poorer HRQoL [OR: 0.64, 95% CI: 0.57–0.71], [OR: 0.63, 95% CI: 0.48–0.82], [OR: 0.67, 95% CI: 0.56–0.80], [OR: 0.69, 95% CI: 0.56–0.86], [OR: 0.55, 95% CI: 0.49–0.62], [OR:0.55, 95% CI: 0.44–0.69], [OR: 0.75, 95% CI: 0.62–0.91] among those with thyroid, colon, lung, cervical, breast, prostate, and ovarian cancer, respectively.

**Discussion:** This study found that income range was associated with HRQoL among cancer survivors. It is plausible that financial resources may lessen the overall burden of cancer survivors, which could improve health-related quality of life among cancer survivors.

## Introduction

When the quality of life is considered in the context of disease and health, it is commonly referred to as health-related quality of life (HRQoL). Health-related quality of life is multidimensional and is composed of, at a minimum, self-perceived health status, physical functioning, and psychological well-being (1). According to the Centers for Disease Control and Prevention (CDC), HRQoL is defined as “an individual's or group's perceived physical and mental health over time” (2). Despite the potentially subjective nature of self-reporting, HRQoL measures tend to reflect the true extent of disability and dysfunction associated with a chronic disease like cancer (3, 4). Due to the significance in both clinical and survivorship contexts, it is of interest to examine potential associations of HRQoL with various sociodemographic and clinical factors.

Thanks to the early diagnosis of cancer and the advancements in technologies and treatments for cancer, the number of cancer survivors has increased significantly over the past decade. However, there are associated negative consequences associated with longer survival time. For example, because of the high cost associated with advanced treatment, patients with cancer can face serious financial challenges (5). Many cancer survivors will return to the workforce while they will encounter higher insurance premiums or co-payment due to “pre-existing conditions” (6, 7). Evidence indicates that cancer survivors carry a greater burden of medically-related financial responsibility, generally known as “financial toxicity,” compared with individuals without a history of cancer (8).

The current study utilized nationally representative data to examine demographic and socioeconomic characteristics and three domains of HRQoL among cancer survivors in the United States (US). We hypothesized that cancer survivors with lesser economic opportunity and thus experienced more financial toxicity, irrespective of cancer site, are more likely to experience poorer HRQoL compared to survivor counterparts with greater economic opportunity.

## Materials and Methods

### Design and Participants

Data were from the 2009 Behavioral Risk Factor Surveillance System (BRFSS) cross-sectional survey conducted by the CDC (9). BRFSS is a population-based, random-digit-dialed telephone survey of the non-institutionalized United States (US) adult population aged ≥18. Standard questions asked by all states query participants on current health-related perceptions (i.e., self-perceived health status), conditions (e.g., diabetes, cardiovascular disease), and behaviors (e.g., tobacco use), as well as demographic characteristics (9). Typically, the “Cancer Survivorship” module is an optional component of the survey. However, in 2009, the module was administered as a standard or required component of the survey (9). Data were analyzed to examine factors associated with reporting multiple measures of HRQoL among all participants who reported having ever been diagnosed with one of seven selected cancer sites. Seven cancer sites were selected based on group sample size, prevalence, and to capture various prognoses. A total of 26,391 survivors were included and grouped according to their reported cancer site. This study was determined as non-human subject research by the University of Arkansas for Medical Sciences Institutional Review Board because we used the de-identified public use data for our analysis.

### Measures

In this survey, HRQoL was measured across the following domains: self-perceived health status, the quantity of poor physical health days per month, and poor mental health days per month. All participants who reported having ever been diagnosed with cancer were asked the following questions:

  “*Would you say that in general your health is…?”*

  “*Would you say that in general your health is; excellent, very good, good, fair, or poor?”*,

  “*Now thinking about your physical health, which includes physical illness and injury, for how many days during the past 30 days was your physical health not good?”* and

  “*Now thinking about your mental health, which includes stress, depression, and problems with emotions, for how many days during the past 30 days was your mental health not good?”* (3)

Responses to the first question were reported as a nominal response (i.e., “excellent” = 1, “very good” = 2, “good” = 3, “fair” = 4, and “poor” = 5). Responses to the second and third questions were reported as a quantity ranging from “0” to “30” (days per month). Dichotomous poor physical and mental health status was defined as having 14 or more days of poor health days (Zhao G, Okoro CA, Hsia J, Town M 2018) (Measuring Health Days CDC 2000).

### Statistical Analysis

Univariate analyses yielded frequencies of sociodemographic characteristics (e.g., sex, race and ethnicity, marital status, educational attainment, annual household income, and health care coverage status) of survivors by cancer site. Group sample size, mean age at the time of the survey, and respective standard deviation was reported by the cancer site. Multivariate analyses yielded the mean self-perceived health status of survivors by site, adjusted for confounders, and calculated with the ordinal 5-point scale of general health using the least square regression method. Confounding variables for all multivariate analyses included age, sex, race and ethnicity, marital status, educational attainment, annual household income, health care coverage, and a history of myocardial infarction, stroke, and/or diabetes. Multivariate logistic regression modeling yielded the odds of reporting “poor” self-perceived health status, more than 14 days or 2 weeks per month of bad physical health days and more than 2 weeks per month of bad mental health days among survivors of the seven selected cancer sites according to income range. Odds ratios and respective 95% confidence intervals (CIs) were reported. If 95% CIs did not contain the null hypothesis value of 1.0, the results were considered to be statistically significant. All statistical analyses were performed using SAS software, version 9.4 (SAS Institute, Inc., Cary, NC). BRFSS utilizes an iterative proportional fitting method in determining the appropriate weights. Therefore, sampling weights from BRFSS were used to calculate the estimated population size and 95% confidence interval (CI).

## Results

### Univariate Analyses

With the consideration of sampling weights from BRFSS, 59,173 were considered having ever been diagnosed with cancer out of the 432,607 participants who completed the survey (Table 1: Sociodemographic Characteristics by Cancer Site). Of the 59,173 survivors, 1,195 had been diagnosed with thyroid cancer, 3,074 with colon cancer, 3,526 with cervical cancer, 10,314 with breast cancer, 5,723 with prostate cancer, and 1,307 with ovarian cancer. The mean age was 59, 71, 69, 54, 67, 72, and 60 years for survivors of thyroid, colon, lung, cervical, breast, prostate, and ovarian cancer, respectively (Table 1). Excluding the sex-specific cancer sites, the majority of participants were female (82, 59, 62%) among thyroid, colon, and lung cancer survivors, respectively. Non-Hispanic Whites were the majority race-ethnicity across all seven cancer sites. Level of educational attainment among survivors varied by cancer site, although most had at least graduated high school. An annual household income of < $50,000 was most common among survivors across all seven cancer sites. Participants widely “refused” or responded as “unsure” when asked if they had health care coverage (Table 1).

**Table 1:** Sociodemographic characteristics of survivors of various cancers.

	**Thyroid**	**Colon**	**Lung**	**Cervical**	**Breast**	**Prostate**	**Ovarian**
*N* (%)	1,195 (2.0)	3,074 (5.2)	1,252 (2.1)	3,512 (6.0)	10,314 (17.6)	5,713 (9.8)	1,304 (2.2)
Age (mean, SD)	59.1 (13.9)	70.5 (12.8)	68.9 (11.7)	53.7 (15.2)	67.4 (13.2)	72.2 (9.6)	60.2 (15.5)
**Sex**							
Female	979 (81.9)	1,810 (58.9)	772 (61.7)	3,512 (99.6)	10,248 (99.4)		1,304 (99.8)
Male	216 (18.1)	1,264 (41.1)	480 (38.3)		66 (0.6)	5,713 (99.8)	
**Race, ethnicity**							
White only, non-hispanic	1,018 (85.2)	2,597 (84.5)	1,073 (85.7)	2,867 (81.3)	8,801 (85.3)	4,713 (82.4)	1,067 (81.6)
Black only, non-hispanic	55 (4.6)	210 (6.8)	86 (6.9)	202 (5.7)	638 (6.2)	523 (9.1)	80 (6.1)
Hispanic	62 (5.2)	109 (3.6)	26 (2.1)	194 (5.5)	355 (3.4)	193 (3.4)	62 (4.7)
Other race only, non-hispanic	41 (3.4)	66 (2.2)	33 (2.6)	138 (3.9)	275 (2.7)	148 (2.6)	43 (3.3)
Multi-racial, non-hispanic	12 (1.0)	49 (1.6)	20 (1.6)	106 (3.0)	160 (1.6)	72 (1.3)	46 (3.5)
Marital status							
Married	715 (59.8)	1,479 (48.1)	560 (44.7)	1,510 (42.8)	4,642 (45)	3,877 (67.7)	558 (42.7)
Other	480 (40.2)	1,595 (51.9)	692 (55.3)	2,016 (57.2)	5,672 (55)	1,846 (32.3)	749 (57.3)
**Education**							
Less than high school	66 (5.5)	405 (13.2)	212 (16.9)	424 (12.0)	832 (8.1)	646 (11.3)	159 (12.2)
High school	336 (28.1)	1,057 (34.4)	468 (37.4)	1,164 (33.0)	3,248 (31.5)	1,566 (27.4)	429 (32.8)
Some college	356 (29.8)	816 (26.6)	326 (26.0)	1,169 (33.2)	2,962 (28.7)	1,241 (21.7)	375 (28.7)
College	432 (36.2)	786 (25.6)	242 (19.3)	764 (21.7)	3,258 (31.6)	2,263 (39.5)	341 (26.1)
**Income**							
<$15,000	114 (9.5)	403 (13.1)	190 (15.2)	666 (18.9)	1,159 (11.2)	397 (6.9)	227 (17.4)
$15,000 to <25,000	173 (14.5)	666 (21.7)	279 (22.3)	743 (21.1)	1,946 (18.9)	917 (16.0)	295 (22.6)
$25,000 to <35,000	112 (9.4)	401 (13.0)	189 (15.1)	400 (11.3)	1,287 (12.5)	754 (13.2)	132 (10.1)
$35,000 to <50,000	144 (12.1)	408 (13.3)	151 (12.1)	449 (12.7)	1,392 (13.5)	959 (16.8)	173 (13.2)
$50,000 or more	502 (42.0)	709 (23.1)	223 (17.8)	890 (25.2)	2,755 (26.7)	2,091 (36.5)	293 (22.4)
**Health care coverage**							
Have health care coverage	700 (58.6)	796 (25.9)	359 (28.7)	2,147 (60.9)	3,605 (35.0)	1,060 (18.5)	640 (49.0)
Do not have health care coverage	54 (4.5)	75 (2.4)	28 (2.2)	506 (14.4)	332 (3.2)	84 (1.5)	138 (10.6)
Don't know/not sure/refused	441 (36.9)	2,203 (71.7)	865 (69.1)	873 (24.8)	6,377 (61.8)	4,579 (80.0)	529 (40.4)

### Multivariate Analyses

**Figure 1:**
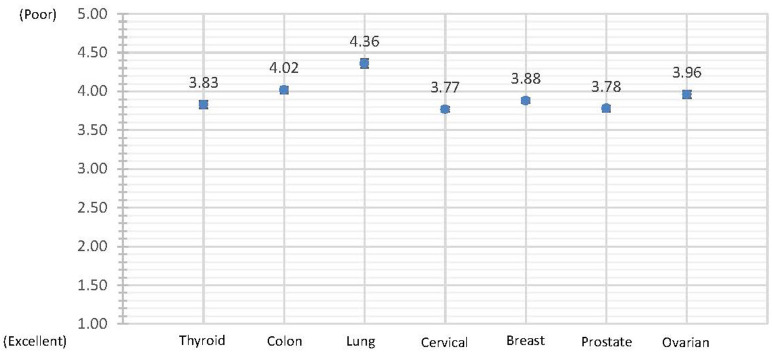
Adjusted mean self-perceived health status. Self-Perceived Health is reported as a whole value on a 5-point scale where “Excellent” = 1; “Very Good” = 2; “Good” = 3; “Fair” = 4; and “Poor” = 5.

Adjusted mean self-perceived health status among survivors of thyroid, colon, lung, cervical, breast, prostate, and ovarian cancer was 3.83 ± 0.05, 4.02 ± 0.04, 4.36 ± 0.06, 3.77 ± 0.03, 3.88 ± 0.03, 3.78 ± 0.04, and 3.96 ± 0.05, respectively (Figure 1: Adjusted Mean Self-Perceived Health Status). A positive dose-response effect was observed between the income range and all three HRQoL measures across all seven cancer sites (Table 2: Odds of Reporting Poor HRQoL).

**Table 2:** Odds of reporting poor health-related quality of life among cancer survivors.

**Cancer Site**	**Income**	**OR^1^ [95% CI^ȧ^^1^]**	**OR^2^ [95% CI^ȧ^^2^]**	**OR^3^ [95% CI^ȧ^^3^]**
Thyroid	<$15,000^*^	Ref	Ref	Ref
*n* = 1,195	$15,000 to <25,000	0.30 [0.11, 0.81]	0.56 [0.22, 1.42]	0.61 [0.22, 1.70]
	$25,000 to <35,000	0.30 [0.09, 1.06]	0.53 [0.16, 1.73]	0.37 [0.11, 1.26]
	$35,000 to <50,000	0.14 [0.04, 0.51]	0.45 [0.16, 1.24]	0.50 [0.16, 1.53]
	$50,000 or more	0.13 [0.04, 0.40]	0.21 [0.08, 0.57]	0.51 [0.18, 1.46]
Colon	<$15,000	Ref	Ref	Ref
*n* = 3,074	$15,000 to <25,000	0.64 [0.35, 1.17]	0.64 [0.32, 1.28]	0.85 [0.39, 1.85]
	$25,000 to <35,000	0.39 [0.18, 0.86]	0.59 [0.25, 1.40]	0.36 [0.13, 1.02]
	$35,000 to <50,000	0.33 [0.15, 0.69]	0.54 [0.25, 1.19]	0.58 [0.24, 1.37]
	$50,000 or more	0.19 [0.09, 0.40]	0.20 [0.09, 0.42]	0.43 [0.18, 1.01]
Lung	<$15,000	Ref	Ref	Ref
*n* = 1,252	$15,000 to <25,000	0.48 [0.22, 1.03]	0.40 [0.15, 1.07]	0.54 [0.15, 1.90]
	$25,000 to <35,000	0.36 [0.14, 0.89]	1.7 [0.46, 5.95]	0.58 [0.14, 2.33]
	$35,000 to <50,000	0.30 [0.11, 0.78]	0.22 [0.06, 0.72]	0.09 [0.02, 0.55]
	$50,000 or more	0.20 [0.08, 0.50]	0.41 [0.13, 1.24]	0.05 [0.01, 0.34]
Cervical	<$15,000	Ref	Ref	Ref
*n* = 3,512	$15,000 to <25,000	0.44 [0.31, 0.63]	0.53 [0.38, 0.74]	0.74 [0.53, 1.03]
	$25,000 to <35,000	0.21 [0.13, 0.35]	0.36 [0.23, 0.55]	0.40 [0.26, 0.60]
	$35,000 to <50,000	0.18 [0.11, 0.30]	0.22 [0.14, 0.34]	0.28 [0.18, 0.43]
	$50,000 or more	0.09 [0.06, 0.16]	0.19 [0.12, 0.28]	0.27 [0.18, 0.39]
Breast	<$15,000	Ref	Ref	Ref
*n* = 10,314	$15,000 to <25,000	0.49 [0.34, 0.70]	0.52 [0.36, 0.75]	0.69 [0.47, 1.02]
	$25,000 to <35,000	0.28 [0.18, 0.44]	0.35 [0.23, 0.54]	0.31 [0.20, 0.50]
	$35,000 to <50,000	0.21 [0.13, 0.33]	0.29 [0.19, 0.43]	0.30 [0.19, 0.46]
	$50,000 or more	0.16 [0.10, 0.25]	0.20 [0.13, 0.30]	0.23 [0.15, 0.35]
Prostate	<$15,000	Ref	Ref	Ref
*n* = 5,713	$15,000 to <25,000	1.70 [0.70, 4.10]	0.99 [0.40, 2.48]	0.41 [0.12, 1.34]
	$25,000 to <35,000	0.54 [0.20, 1.52]	0.52 [0.20, 1.41]	0.14 [0.04, 0.53]
	$35,000 to <50,000	0.41 [0.15, 1.13]	0.40 [0.16, 0.99]	0.32 [0.11, 0.95]
	$50,000 or more	0.14 [0.05, 0.39]	0.36 [0.15, 0.88]	0.19 [0.06, 0.57]
Ovarian	<$15,000	Ref	Ref	Ref
*n* = 1,304	$15,000 to <25,000	0.33 [0.17, 0.64]	0.67 [0.36, 1.25]	0.40 [0.20, 0.81]
	$25,000 to <35,000	0.38 [0.16, 0.89]	0.42 [0.19, 0.95]	0.47 [0.20, 1.13]
	$35,000 to <50,000	0.33 [0.14, 0.75]	0.30 [0.14, 0.68]	0.14 [0.06, 0.35]
	$50,000 or more	0.27 [0.13, 0.60]	0.39 [0.19, 0.81]	0.20 [0.09, 0.45]

1, 2, 3 *Adjusted odds ratio*.

∧ *95% Confidence Interval*.

* *Reference Group*. *OR^1^ and 95% CI^∧^^1^: Odds of reporting “poor” self-perceived health status*. *OR^2^ and 95% CI^∧^^2^: Odds of reporting more than 2 weeks per month of bad physical health days*. *OR^3^ and 95% CI^∧^^3^: Odds of reporting more than 2 weeks per month of bad mental health days*.

### Self-Perceived Health Status

Univariate analysis was conducted to examine the relationship between socieodemographic charcateristics and self-perceived general health, poor physical health, and poor mental health among cancer survivors of seven cancer sites included. We found statistically significant association in every factors (Supplementary Table 1). However, income was consistently and inversely associated with a higher chance of reporting poorer self-perceived health status. Among survivors of cervical, breast, and ovarian cancers, odds ratio estimates demonstrated a consistent positive-dose response effect, and respective 95% CIs were statistically significant for every range of income.

#### Cervical

Among cervical cancer survivors, the odds of reporting poorer self-perceived health status decreased as income increased [OR: 0.44, 95% CI: 0.31, 0.63], [OR: 0.21, 95% CI: 0.13, 0.35], [OR: 0.18, 95% CI: 0.11, 0.30], [OR: 0.09, 95% CI: 0.06, 0.16] among those with an annual household income range of <$15,000 (ref), $15,000 to <25,000, $25,000 to <35,000, $35,000 to <50,000, and $50,000 or more, respectively (Table 2).

#### Breast

Among breast cancer survivors, the odds of reporting poorer self-perceived health status decreased as income increased [OR: 0.49, 95% CI: 0.34, 0.70], [OR: 0.28, 95% CI: 0.18, 0.44], [OR: 0.21, 95% CI: 0.13, 0.33], [OR: 0.16, 95% CI: 0.10, 0.25] among those with an annual household income range of <$15,000 (ref), $15,000 to <25,000, $25,000 to <35,000, $35,000 to <50,000, and $50,000 or more, respectively (Table 2).

#### Ovarian

Among ovarian cancer survivors, the odds of reporting poorer self-perceived health status decreased as income increased [OR: 0.33, 95% CI: 0.17, 0.64], [OR: 0.38, 95% CI: 0.16, 0.89], [OR: 0.33, 95% CI: 0.14, 0.75], [OR: 0.27, 95% CI: 0.13, 0.60] among those with an annual household income range of <$15,000 (ref), $15,000 to <25,000, $25,000 to <35,000, $35,000 to <50,000, and $50,000 or more, respectively (Table 2).

### Physical Health

Income was inversely associated with a higher chance for reporting two or more weeks of bad physical health days per month. A positive-dose response was observed, albeit, less consistently across cancer sites compared to self-perceived health status. Among survivors of cervical and breast cancers, odds ratio estimates demonstrated a consistent positive-dose response effect, and respective 95% CIs were statistically significant for every range of income.

#### Cervical

Among cervical cancer survivors, the odds of reporting two or more weeks per month of bad physical health days decreased as income increased [OR: 0.33, 95% CI: 0.17, 0.64], [OR: 0.38, 95% CI: 0.16, 0.89], [OR: 0.33, 95% CI: 0.14, 0.75], [OR: 0.27, 95% CI: 0.13, 0.60] among those with an annual household income range of <$15,000 (ref), $15,000 to <25,000, $25,000 to <35,000, $35,000 to <50,000, and $50,000 or more, respectively (Table 2).

#### Breast

Among breast cancer survivors, the odds of reporting two or more weeks per month of bad physical health days decreased as income increased [OR: 0.33, 95% CI: 0.17, 0.64], [OR: 0.38, 95% CI: 0.16, 0.89], [OR: 0.33, 95% CI: 0.14, 0.75], [OR: 0.27, 95% CI: 0.13, 0.60] among those with an annual household income range of <$15,000 (ref), $15,000 to <25,000, $25,000 to <35,000, $35,000 to <50,000, and $50,000 or more, respectively (Table 2).

### Mental Health

Income was often inversely associated with a higher chance for reporting two or more weeks of bad mental health days per month. A positive dose-response effect between income and poor mental health days was observed among survivors of lung, cervical, and breast cancers. However, CIs were not consistently statistically significant for all ranges of income.

#### Lung

Among lung cancer survivors, the odds of reporting two or more weeks per month of bad mental health days decreased as income increased [OR: 0.33, 95% CI: 0.17, 0.64], [OR: 0.38, 95% CI: 0.16, 0.89], [OR: 0.33, 95% CI: 0.14, 0.75], [OR: 0.27, 95% CI: 0.13, 0.60] among those with an annual household income range of <$15,000 (ref), $15,000 to <25,000, $25,000 to <35,000, $35,000 to <50,000 and $50,000 or more, respectively (Table 2).

#### Cervical

Among cervical cancer survivors, the odds of reporting two or more weeks per month of bad mental health days decreased as income increased [OR: 0.33, 95% CI: 0.17, 0.64], [OR: 0.38, 95% CI: 0.16, 0.89], [OR: 0.33, 95% CI: 0.14, 0.75], [OR: 0.27, 95% CI: 0.13, 0.60] among those with an annual household income range of <$15,000 (ref), $15,000 to <25,000, $25,000 to <35,000, $35,000 to <50,000, and $50,000 or more, respectively (Table 2).

#### Breast

Among breast cancer survivors, the odds of reporting two or more weeks per month of bad mental health days decreased as income increased [OR: 0.33, 95% CI: 0.17, 0.64], [OR: 0.38, 95% CI: 0.16, 0.89], [OR: 0.33, 95% CI: 0.14, 0.75], [OR: 0.27, 95% CI: 0.13, 0.60] among those with an annual household income range of <$15,000 (ref), $15,000 to <25,000, $25,000 to <35,000, $35,000 to <50,000, and $50,000 or more, respectively (Table 2).

## Discussion

This study utilized the latest national representative survey data to examine factors associated with HRQoL. We found that lower family income is the primary factor associated with both poorer mental and physical health among cancer survivors, regardless of the cancer site. Our finding is consistent with the analysis of the BRFSS survey from 2000 to 2002 (10). Another study used a more recent BRFSS examined the HRQoL among cancer survivors utilized the cancer survivor module as our study (11). However, the study of the 2016 BRFSS survey 2016 only included nine states in the US. Although the authors did not have household income levels in their models, they found non-employment status is significantly associated with all measures of HRQoL, which is related to the financial well-being of the cancer survivor. Female and marital status of divorced/widowed/separated/never married are the only other factors associated with all measures of HRQoL. Our analysis did not find a statistically significant association of gender and marital status after adjusting for confounders.

Very few studies evaluated sociodemographic characteristics for cancer survivors on HRQoL across various cancer sites. The publication by Applewhite summarized published studies of the quality of life among survivors of the thyroid, colon, glioma, breast, and gynecologic cancer. The authors suggested that breast cancer survivors had a better overall quality of life than all other cancers compared. The overall quality of life was similar among patients with colon cancer, glioma, gynecologic cancer, and thyroid cancer (12). Our study, however, found that. regardless of cancer site, income level was inversely associated with HRQoL among cancer survivors. We do not see a significant racial difference among different racial groups either. It is plausible that financial resources may lessen the overall burden of cancer survivors, which could improve self-perceived health-related quality of life, psychological well-being, and physical function among cancer survivors. We believe the current study's findings add to a growing body of literature demonstrating that survivorship is associated with financial hardship (8, 13–21).

Cancer survivors are living longer with their cancer as a chronic illness, thanks to early diagnosis and advancements in medical technologies and treatment (22). Cancer survivors have to be monitored for an extensive period of time (23). Therefore, there is an increased reliance on patients to make larger co-payments and financial contributions to their healthcare. It will result in financial toxicity results when medical expenditures with associated out-of-pocket costs are high relative to family income. Research has demonstrated that financial toxicities appear to constitute part of the pathway that ultimately leads to adverse health outcomes and poorer HRQoL (24–26). Even in countries where there is universal healthcare or when individuals have health insurance, additional patient out-of-pocket expenses are expected (22, 27). Chen et al. reported that an income gradient in avoidable mortality rates persisted throughout a 40-year study period from 1971 to 2008 using national data of all deaths reported in Taiwan (28). Universal guaranteed access to medical care in 1995 may have helped reduce, but did not eliminate, the income gradient in mortality disparities. Income vulnerability also adversely impacts the utilization of healthcare services (29).

Studies found that younger and minority cancer patients are disproportionately affected by financial toxicity as they may have fewer savings, more educational debts, and fewer assets than older cancer patients (30, 31). Because these younger cancer patients are likely still active in the workforce. Doctor visits, appointments for exams and treatments, the time needed to recover from treatment, and follow-up visits can all make it difficult to take time away from their careers (32). Psychological stress for an extended period could have a toll on both their physical and mental well-being (33, 34). We found that increased family income level was significantly associated with fewer bad physical days among cancer survivors of six sites after adjusting for confounders, including age, other than lung cancer, with a clear dose-response relationship. Higher family income was associated with fewer bad mental days among survivors of lung, cervical, breast, prostate, and ovarian cancers. The financial ability to access resources to address both mental and physical stress appeared to play a significant role in the well-being of cancer survivors, regardless of the type of cancer. We did not find a significant association between the age of participants and HRQoL in any cancer.

Mental and physical health among people living with and beyond cancer has been identified as a growing clinical and research priority (35, 36). This study provides a cross-sectional examination of the factors associated with HRQoL, which including both mental and physical health, among cancer survivors using a national representative sample. However, like many others, this study has its limitations such that the results should be interpreted with consideration of its design. First, the cross-sectional nature of this survey yields the possibility of survivorship bias. The length of time that has passed since their last treatment is unknown for each survivor. Cancer survivors in the survey were likely diagnosed at an earlier stage and were healthy enough to complete the survey. Additionally, the selection of seven pathologically heterogeneous cancer sites might introduce questions concerning disparities in treatment toxicity (e.g., surgery vs. chemotherapy and radiation), economic burden (e.g., duration and extent of treatment), and lifetime prognoses (e.g., survival times differ markedly).

## Conclusion

The survey was conducted prior to the enactment of the Affordable Care Act (ACA). Ideally, the health care reform would have eased the contribution of family income to the HRQoL among cancer survivors. However, the study conducted in Taiwan did not observe reversing the trend for the relationship between the financial burden for cancer survivors and HRQoL after the implementation of universal guaranteed access to medical care in Taiwan (28). It has been more than 10 years since BRFSS has included the module of HRQoL among cancer survivors in all 50 states and Washington, DC. Public health researchers and policymakers need the information to assess the impact of the ACA on the HRQoL among cancer survivors regarding financial well-being. We hope the CDC will consider implementing the cancer survivor module in all states in the coming BRFSS survey.

## Data Availability Statement

Publicly available datasets were analyzed in this study. This data can be found here: https://www.cdc.gov/brfss/annual_data/annual_2009.htm.

## Author Contributions

LS initiated the research concept, participated in the data analysis, and completed the draft of manuscript. SO'C conducted the statistical analysis and initiated the manuscript. T-CC contributed in the draft and organization of the manuscript. All authors participated in the review and revision of the manuscript.

## Funding

This study was made possible with the funding support from University of Arkansas for Medical Sciences Winthrop P. Rockefeller Cancer Institute.

## Conflict of Interest

The authors declare that the research was conducted in the absence of any commercial or financial relationships that could be construed as a potential conflict of interest.

## Publisher's Note

All claims expressed in this article are solely those of the authors and do not necessarily represent those of their affiliated organizations, or those of the publisher, the editors and the reviewers. Any product that may be evaluated in this article, or claim that may be made by its manufacturer, is not guaranteed or endorsed by the publisher.

## References

[1] Palermo TM Long AC Lewandowski AS Drotar D Quittner AL Walker LS. Evidence-based assessment of health-related quality of life and functional impairment in pediatric psychology. J Pediatr Psychol. (2008) 33:983–96; discussion 997–1088. 10.1093/jpepsy/jsn038 18430762 PMC2543105

[2] Centers for Disease Control and Prevention. Measuring Healthy Days: Population Assessment of Health-Related Quality of Life: Population Assessment of Health-Related Quality of Life. Atlanta, GA: U.S. Department of Health and Human Services, Centers for Disease Control and Prevention National Center for Chronic Disease Prevention and Health Promotion Division of Adult and Community Health (2000).

[3] Cella D Nowinski CJ. Measuring quality of life in chronic illness: the functional assessment of chronic illness therapy measurement system. Arch Phys Med Rehabil. (2002) 83 (12 Suppl 2):S10–7. 10.1053/apmr.2002.36959 12474167

[4] Megari K. Quality of life in chronic disease patients. Health Psychol Res. (2013) 1:e27. 10.4081/hpr.2013.e27 26973912 PMC4768563

[5] Gordon LG Merollini KMD Lowe A Chan RJ. A systematic review of financial toxicity among cancer survivors: we can't pay the co-pay. Patient. (2017) 10:295–309. 10.1007/s40271-016-0204-x 27798816

[6] Pak TY Kim H Kim KT. The long-term effects of cancer survivorship on household assets. Health Econ Rev. (2020) 10:2. 10.1186/s13561-019-0253-7 31933035 PMC6958665

[7] Sanford NN Lam MB Butler SS Ahn C Beg MS Aizer AA. Self-reported reasons and patterns of noninsurance among cancer survivors before and after implementation of the affordable care act, 2000-2017. JAMA Oncol. (2019) 5:e191973. 10.1001/jamaoncol.2019.1973 31091534 PMC6537771

[8] Zheng Z Jemal A Han X Guy GP Jr Li C Davidoff AJ. Medical financial hardship among cancer survivors in the United States. Cancer. (2019) 125:1737–47. 10.1002/cncr.31913 30663039

[9] Centers for Disease Control and Prevention. Behavioral Risk Factor Surveillance System Survey Questionnaire. In: Atlanta, GA: U.S. Department of Health and Human Services (2009).

[10] Richardson LC Wingo PA Zack MM Zahran HS King JB. Health-related quality of life in cancer survivors between ages 20 and 64 years: population-based estimates from the Behavioral Risk Factor Surveillance System. Cancer. (2008) 112:1380–9. 10.1002/cncr.23291 18219664

[11] Cox-Martin E Anderson-Mellies A Borges V Bradley C. Chronic pain, health-related quality of life, and employment in working-age cancer survivors. J Cancer Surviv. (2020) 14:179–87. 10.1007/s11764-019-00843-0 31828603 PMC7473420

[12] Applewhite MK James BC Kaplan SP Angelos P Kaplan EL Grogan RH. Quality of life in thyroid cancer is similar to that of other cancers with worse survival. World J Surg. (2016) 40:551–61. 10.1007/s00268-015-3300-5 26546191

[13] Ekwueme DU Zhao J Rim SH de Moor JS Zheng Z Khushalani JS. Annual out-of-pocket expenditures and financial hardship among cancer survivors aged 18-64 years - United States, 2011-2016. Morb Mortal Wkly Rep. (2019) 68:494–9. 10.15585/mmwr.mm6822a2 31170127 PMC6553808

[14] Yabroff KR Zhao J Zheng Z Rai A Han X. Medical financial hardship among cancer survivors in the united states: what do we know? What do we need to know? Cancer Epidemiol Biomarkers Prev. (2018) 27:1389–97. 10.1158/1055-9965.EPI-18-0617 30429132

[15] Kunos CA Abdallah R. Financial toxicity encountered in therapeutic radiopharmaceutical clinical development for ovarian cancer. Pharmaceuticals. (2020) 13:181. 10.3390/ph13080181 32764223 PMC7464475

[16] Cook EE Rosenberg SM Ruddy KJ Barry WT Greaney M Ligibel J. Prospective evaluation of the impact of stress, anxiety, and depression on household income among young women with early breast cancer from the Young and Strong trial. BMC Public Health. (2020) 20:1514. 10.1186/s12889-020-09562-z 33023562 PMC7541223

[17] McDougall JA Anderson J Adler Jaffe S Guest DD Sussman AL Meisner ALW. Food insecurity and forgone medical care among cancer survivors. JCO Oncol Pract. (2020) 16:e922–32. 10.1200/JOP.19.00736 32384017 PMC7489488

[18] Gupta SK Mazza MC Hoyt MA Revenson TA. The experience of financial stress among emerging adult cancer survivors. J Psychosoc Oncol. (2020) 38:435–48. 10.1080/07347332.2019.1707928 31983313 PMC7316584

[19] Han X Zhao J Zheng Z de Moor JS Virgo KS Yabroff KR. Medical financial hardship intensity and financial sacrifice associated with cancer in the United States. Cancer Epidemiol Biomarkers Prev. (2020) 29:308–17. 10.1158/1055-9965.EPI-19-0460 31941708 PMC7007367

[20] Odahowski CL Zahnd WE Zgodic A Edward JS Hill LN Davis MM. Financial hardship among rural cancer survivors: An analysis of the Medical Expenditure Panel Survey. Prev Med. (2019) 129S:105881. 10.1016/j.ypmed.2019.105881 PMC7190004 31727380

[21] Barrows CE Belle JM Fleishman A Lubitz CC James BC. Financial burden of thyroid cancer in the United States: An estimate of economic and psychological hardship among thyroid cancer survivors. Surgery. (2020) 167:378–84. 10.1016/j.surg.2019.09.010 31653488

[22] de Souza JA Wong YN. Financial distress in cancer patients. J Med Person. (2013) 11:1–7. 10.1007/s12682-013-0152-3 24349677 PMC3859450

[23] Yabroff KR Lawrence WF Clauser S Davis WW Brown ML. Burden of illness in cancer survivors: findings from a population-based national sample. J Natl Cancer Inst. (2004) 96:1322–30. 10.1093/jnci/djh255 15339970

[24] Zafar SY McNeil RB Thomas CM Lathan CS Ayanian JZ Provenzale D. Population-based assessment of cancer survivors' financial burden and quality of life: a prospective cohort study. J Oncol Pract. (2015) 11:145–50. 10.1200/JOP.2014.001542 25515717 PMC4371118

[25] Lathan CS Cronin A Tucker-Seeley R Zafar SY Ayanian JZ Schrag D. Association of financial strain with symptom burden and quality of life for patients with lung or colorectal cancer. J Clin Oncol. (2016) 34:1732–40. 10.1200/JCO.2015.63.2232 26926678 PMC4966336

[26] Perrone F Jommi C Di Maio M Gimigliano A Gridelli C Pignata S. The association of financial difficulties with clinical outcomes in cancer patients: secondary analysis of 16 academic prospective clinical trials conducted in Italy. Ann Oncol. (2016) 27:2224–9. 10.1093/annonc/mdw433 27789469

[27] Laba TL Essue BM Jan S. Financing options to sustain Medicare: are we committed to universalism? Med J Aust. (2015) 203:244–5.e241. 10.5694/mja15.00431 26377286

[28] Chen BK Yang YT Yang CY. Trends in amenable deaths based on township income quartiles in Taiwan, 1971-2008: did universal health insurance close the gap? J Public Health. (2016) 38:e524–36. 10.1093/pubmed/fdv156 28158683

[29] Chen BK Hibbert J Cheng X Bennett K. Travel distance and sociodemographic correlates of potentially avoidable emergency department visits in California, 2006-2010: an observational study. Int J Equity Health. (2015) 14:30. 10.1186/s12939-015-0158-y 25889646 PMC4391132

[30] Yabroff KR Dowling EC Guy GP Jr Banegas MP Davidoff A Han X. Financial hardship associated with cancer in the united states: findings from a population-based sample of adult cancer survivors. J Clin Oncol. (2016) 34:259–67. 10.1200/JCO.2015.62.0468 26644532 PMC4872019

[31] Pisu M Kenzik KM Oster RA Drentea P Ashing KT Fouad M. Economic hardship of minority and non-minority cancer survivors 1 year after diagnosis: another long-term effect of cancer? Cancer. (2015) 121:1257–64. 10.1002/cncr.29206 25564986 PMC4393356

[32] The American Cancer Society Medical Editorial Content Team. Special Issues for Young Adults With Cancer. American Cancer Society (2019). Available online at: https://www.cancer.org/cancer/cancer-in-young-adults/special-issues.html (accessed June 14, 2020).

[33] Mariotti A. The effects of chronic stress on health: new insights into the molecular mechanisms of brain-body communication. Future Sci OA. (2015) 1:FSO23. 10.4155/fso.15.21 28031896 PMC5137920

[34] Naughton MJ Weaver KE. Physical and mental health among cancer survivors: considerations for long-term care and quality of life. N C Med J. (2014) 75:283–6. 10.18043/ncm.75.4.283 25046097 PMC4503227

[35] Niedzwiedz CL Knifton L Robb KA Katikireddi SV Smith DJ. Depression and anxiety among people living with and beyond cancer: a growing clinical and research priority. BMC Cancer. (2019) 19:943. 10.1186/s12885-019-6181-4 31604468 PMC6788022

[36] Elshahat S Treanor C Donnelly M. Factors influencing physical activity participation among people living with or beyond cancer: a systematic scoping review. Int J Behav Nutr Phys Act. (2021) 18:50. 10.1186/s12966-021-01116-9 33823832 PMC8025326

