# Supplementary material for: Association Between Household Income and Self-Perceived Health Status and Poor Mental and Physical Health Among Cancer Survivors
Source: Front Public Health. 2021 Dec 7;9:752868. doi: 10.3389/fpubh.2021.752868 (PMC8688689; doi:10.3389/fpubh.2021.752868)
Supplement: Supplementary file 1 [file Data_Sheet_1.PDF]

Table 1. Sociodemographic characteristics of survivors of various cancers

	Thyroid	Colon	Lung	Cervical	Breast	Prostate	Ovarian
N (%)	1195 (2.0)	3074 (5.2)	1252 (2.1)	3512 (6.0)	10314 (17.6)	5713 (9.8)	1304 (2.2)
Age (mean, SD)	59.1 (13.9)	70.5 (12.8)	68.9 (11.7)	53.7 (15.2)	67.4 (13.2)	72.2 (9.6)	60.2 (15.5)
**Sex**							
Female	979 (81.9)	1810 (58.9)	772 (61.7)	3512 (99.6)	10248 (99.4)		1304 (99.8)
Male	216 (18.1)	1264 (41.1)	480 (38.3)		66 (0.6)	5713 (99.8)	
**Race, Ethnicity**							
White only, Non-Hispanic	1018 (85.2)	2597 (84.5)	1073 (85.7)	2867 (81.3)	8801 (85.3)	4713 (82.4)	1067 (81.6)
Black only, Non-Hispanic	55 (4.6)	210 (6.8)	86 (6.9)	202 (5.7)	638 (6.2)	523 (9.1)	80 (6.1)
Hispanic	62 (5.2)	109 (3.6)	26 (2.1)	194 (5.5)	355 (3.4)	193 (3.4)	62 (4.7)
Other race only, Non-Hispanic	41 (3.4)	66 (2.2)	33 (2.6)	138 (3.9)	275 (2.7)	148 (2.6)	43 (3.3)
Multi-racial, Non-Hispanic	12 (1.0)	49 (1.6)	20 (1.6)	106 (3.0)	160 (1.6)	72 (1.3)	46 (3.5)
**Marital status**							
Married	715 (59.8)	1479 (48.1)	560 (44.7)	1510 (42.8)	4642 (45)	3877 (67.7)	558 (42.7)
Other	480 (40.2)	1595 (51.9)	692 (55.3)	2016 (57.2)	5672 (55)	1846 (32.3)	749 (57.3)
**Education**							
Less than High School	66 (5.5)	405 (13.2)	212 (16.9)	424 (12.0)	832 (8.1)	646 (11.3)	159 (12.2)
High School	336 (28.1)	1057 (34.4)	468 (37.4)	1164 (33.0)	3248 (31.5)	1566 (27.4)	429 (32.8)
Some College	356 (29.8)	816 (26.6)	326 (26.0)	1169 (33.2)	2962 (28.7)	1241 (21.7)	375 (28.7)
College	432 (36.2)	786 (25.6)	242 (19.3)	764 (21.7)	3258 (31.6)	2263 (39.5)	341 (26.1)
**Income**							
Less than $15,000	114 (9.5)	403 (13.1)	190 (15.2)	666 (18.9)	1159 (11.2)	397 (6.9)	227 (17.4)
$15,000 to less than $25,000	173 (14.5)	666 (21.7)	279 (22.3)	743 (21.1)	1946 (18.9)	917 (16.0)	295 (22.6)
$25,000 to less than $35,000	112 (9.4)	401 (13.0)	189 (15.1)	400 (11.3)	1287 (12.5)	754 (13.2)	132 (10.1)
$35,000 to less than $50,000	144 (12.1)	408 (13.3)	151 (12.1)	449 (12.7)	1392 (13.5)	959 (16.8)	173 (13.2)

$50,000 or more	502 (42.0)	709 (23.1)	223 (17.8)	890 (25.2)	2755 (26.7)	2091 (36.5)	293 (22.4)
**Health care coverage**							
Have health care coverage	700 (58.6)	796 (25.9)	359 (28.7)	2147 (60.9)	3605 (35.0)	1060 (18.5)	640 (49.0)
Do not have health care coverage	54 (4.5)	75 (2.4)	28 (2.2)	506 (14.4)	332 (3.2)	84 (1.5)	138 (10.6)
Don't know/Not sure/Refused	441 (36.9)	2203 (71.7)	865 (69.1)	873 (24.8)	6377 (61.8)	4579 (80.0)	529 (40.4)

Table 2: Odds of Reporting Poor Health-Related Quality of Life among Cancer Survivors

Cancer Site	Income	OR[1] [95% CI^[1]]	OR[2] [95% CI^[2]]	OR[3] [95% CI^[3]]
Thyroid	Less than $15,000*	Ref	Ref	Ref
n = 1195	$15,000 to less than $25,000	0.30 [0.11, 0.81]	0.56 [0.22, 1.42]	0.61 [0.22, 1.70]
	$25,000 to less than $35,000	0.30 [0.09, 1.06]	0.53 [0.16, 1.73]	0.37 [0.11, 1.26]
	$35,000 to less than $50,000	0.14 [0.04, 0.51]	0.45 [0.16, 1.24]	0.50 [0.16, 1.53]
	$50,000 or more	0.13 [0.04, 0.40]	0.21 [0.08, 0.57]	0.51 [0.18, 1.46]
Colon	Less than $15,000	Ref	Ref	Ref
n = 3074	$15,000 to less than $25,000	0.64 [0.35, 1.17]	0.64 [0.32, 1.28]	0.85 [0.39, 1.85]
	$25,000 to less than $35,000	0.39 [0.18, 0.86]	0.59 [0.25, 1.40]	0.36 [0.13, 1.02]
	$35,000 to less than $50,000	0.33 [0.15, 0.69]	0.54 [0.25, 1.19]	0.58 [0.24, 1.37]
	$50,000 or more	0.19 [0.09, 0.40]	0.20 [0.09, 0.42]	0.43 [0.18, 1.01]
Lung	Less than $15,000	Ref	Ref	Ref
n = 1252	$15,000 to less than $25,000	0.48 [0.22, 1.03]	0.40 [0.15, 1.07]	0.54 [0.15, 1.90]
	$25,000 to less than $35,000	0.36 [0.14, 0.89]	1.7 [0.46, 5.95]	0.58 [0.14, 2.33]
	$35,000 to less than $50,000	0.30 [0.11, 0.78]	0.22 [0.06, 0.72]	0.09 [0.02, 0.55]
	$50,000 or more	0.20 [0.08, 0.50]	0.41 [0.13, 1.24]	0.05 [0.01, 0.34]
Cervical	Less than $15,000	Ref	Ref	Ref
n = 3512	$15,000 to less than $25,000	0.44 [0.31, 0.63]	0.53 [0.38, 0.74]	0.74 [0.53, 1.03]
	$25,000 to less than $35,000	0.21 [0.13, 0.35]	0.36 [0.23, 0.55]	0.40 [0.26, 0.60]
	$35,000 to less than $50,000	0.18 [0.11, 0.30]	0.22 [0.14, 0.34]	0.28 [0.18, 0.43]
	$50,000 or more	0.09 [0.06, 0.16]	0.19 [0.12, 0.28]	0.27 [0.18, 0.39]
Breast	Less than $15,000	Ref	Ref	Ref

n = 10314	\$15,000 to less than \$25,000	0.49 [0.34, 0.70]	0.52 [0.36, 0.75]	0.69 [0.47, 1.02]
	\$25,000 to less than \$35,000	0.28 [0.18, 0.44]	0.35 [0.23, 0.54]	0.31 [0.20, 0.50]
	\$35,000 to less than \$50,000	0.21 [0.13, 0.33]	0.29 [0.19, 0.43]	0.30 [0.19, 0.46]
	\$50,000 or more	0.16 [0.10, 0.25]	0.20 [0.13, 0.30]	0.23 [0.15, 0.35]
Prostate	Less than \$15,000	Ref	Ref	Ref
n = 5713	\$15,000 to less than \$25,000	1.70 [0.70, 4.10]	0.99 [0.40, 2.48]	0.41 [0.12, 1.34]
	\$25,000 to less than \$35,000	0.54 [0.20, 1.52]	0.52 [0.20, 1.41]	0.14 [0.04, 0.53]
	\$35,000 to less than \$50,000	0.41 [0.15, 1.13]	0.40 [0.16, 0.99]	0.32 [0.11, 0.95]
	\$50,000 or more	0.14 [0.05, 0.39]	0.36 [0.15, 0.88]	0.19 [0.06, 0.57]
Ovarian	Less than \$15,000	Ref	Ref	Ref
n = 1304	\$15,000 to less than \$25,000	0.33 [0.17, 0.64]	0.67 [0.36, 1.25]	0.40 [0.20, 0.81]
	\$25,000 to less than \$35,000	0.38 [0.16, 0.89]	0.42 [0.19, 0.95]	0.47 [0.20, 1.13]
	\$35,000 to less than \$50,000	0.33 [0.14, 0.75]	0.30 [0.14, 0.68]	0.14 [0.06, 0.35]
	\$50,000 or more	0.27 [0.13, 0.60]	0.39 [0.19, 0.81]	0.20 [0.09, 0.45]

[1, 2, 3] : Adjusted odds ratio
^ 95% Confidence Interval
* Reference Group
OR[1] and 95% CI^[1]: Odds of reporting "poor" self-perceived health status
OR[2] and 95% CI^[2]: Odds of reporting more than two weeks per month of bad physical health days
OR[3] and 95% CI^[3]: Odds of reporting more than two weeks per month of bad mental health days

Supplementary Table 1. Health Related Quality of Life based sociodemographic characteristics of cancer survivors

	General Health			Physical Health			Mental Health		
	Good	Not good	p-value	Good	Not good	p-value	Good	Not good	p-value
N (%)									
Age (mean, SD)	66.3 (13.5)	67.3 (13.4)	< .01	66.9 (13.4)	66.0 (13.7)	< .01	67.6 (13.1)	59.2 (14.5)	< .01
**Sex**									
Female	12,569 (67.5%)	6,066 (32.5%)	0.36	14,443 (69.7%)	4,192 (74.0%)	< .01	16,076 (69.0%)	2,559 (82.6%)	< .01
Male	5,186 (66.9%)	2,570 (33.1%)		6,286 (30.3%)	1,470 (26.0%)		7,218 (31.0%)	538 (17.4%)	
**Race, Ethnicity**									
White only, Non-Hispanic	15,319 (86.3%)	6,817 (78.9%)	< .01	17,562 (84.7%)	4,574 (80.8%)	< .01	19,704 (84.6%)	2,432 (78.5%)	< .01
Black only, Non-Hispanic	1,007 (5.7%)	787 (9.1%)		1,382 (6.7%)	412 (7.3%)		1,572 (6.8%)	222 (7.2%)	
Hispanic	535 (3.0%)	466 (5.4%)		705 (3.4%)	296 (5.2%)		813 (3.5%)	188 (6.1%)	
Other race only, Non-Hispanic	621 (3.5%)	374 (4.3%)		769 (3.7%)	226 (4.0%)		847 (3.6%)	148 (4.8%)	
Multi-racial, Non-Hispanic	273 (3.5%)	192 (2.2%)		311 (1.5%)	154 (2.7%)		358 (1.5%)	107 (3.5%)	
**Marital status**									
Married	9,532 (71.5%)	3,809 (28.5%)	< .01	10,895 (52.6%)	2,446 (43.2%)	< .01	12,093 (51.9%)	1,248 (40.3%)	< .01
Other	8,223 (63.0%)	4,827 (37.0%)		9,834 (47.4%)	3,216 (56.8%)		11,201 (48.1%)	1,849 (59.7%)	
**Education**									
Less than High School	1,181 (6.7%)	1,563 (18.1%)	< .01	1,781 (8.6%)	963 (17.0%)	< .01	2,261 (9.7%)	483 (15.6%)	< .01
High School	5,115 (28.8%)	3,153 (36.5%)		6,352 (30.6%)	1,916 (33.8%)		7,242 (31.1%)	1,026 (33.1%)	
Some College	4,969 (28.0%)	2,276 (26.4%)		5,622 (27.1%)	1,623 (28.7%)		6,288 (27.0%)	957 (30.9%)	
College	6,462 (36.4%)	1,624 (18.8%)		6,936 (33.5%)	1,150 (20.3%)		7,460 (32.0%)	626 (20.2%)	
Don't know/ Refused	28 (0.2%)	20 (0.2%)		38 (0.2%)	10 (0.2%)		43 (0.2%)	5 (0.2%)	
**Income**									
Less than $15,000	1,333 (7.5%)	1,823 (21.1%)	< .01	1,903 (9.2%)	1,253 (22.1%)	< .01	2,364 (10.2%)	792 (25.6%)	< .01
$15,000 to less than $25,000	2,844 (16.0%)	2,175 (25.2%)		3,619 (17.5%)	1,400 (24.7%)		4,286 (18.4%)	733 (23.7%)	
$25,000 to less than $35,000	2,204 (12.4%)	1,071 (12.4%)		2,612 (12.6%)	663 (11.7%)		2,930 (12.6%)	345 (11.1%)	
$35,000 to less than $50,000	2,714 (15.3%)	962 (11.1%)		3,063 (14.8%)	613 (10.8%)		3,356 (14.4%)	320 (10.3%)	
$50,000 or more	6,171 (34.8%)	1,292 (15.0%)		6,535 (31.5%)	928 (16.4%)		6,919 (29.7%)	544 (17.6%)	
Don't know/ Refused	2,489 (14.0%)	1,313 (15.2%)		2,997 (14.5%)	805 (14.2%)		3,439 (14.8%)	363 (11.7%)	

**Health care coverage**									
Have health care coverage	6,478 (36.5%)	2,829 (32.8%)	< .01	7,207 (34.8%)	2,100 (37.1%)	< .01	7,709 (33.1%)	1,598 (51.6%)	< .01
Do not have health care coverage	689 (3.9%)	528 (6.1%)		848 (4.1%)	369 (6.5%)		835 (3.6%)	382 (12.3%)	
Don't know/Not sure/Refused	10,588 (59.6%)	5,279 (61.1%)		14,674 (61.1%)	3,193 (56.4%)		14,750 (63.3%)	1,117 (36.1%)	
